# Evidence-based study on antithrombotic therapy in patients at risk of a stroke with paroxysmal atrial fibrillation

**DOI:** 10.3892/etm.2013.1141

**Published:** 2013-06-05

**Authors:** XINJUN CHEN, RONGHUA WAN, WENLONG JIANG, HUA ZHANG, RUOLONG ZHEN, QUANZHONG YING, WEIZHANG LI, HUIDONG QIAN, FENG WEI, ZHUOWEN XU, JIANJIN TANG, HANDONG LEI, YI ZHOU

**Affiliations:** 1 Department of Cardiology, Affiliated Jiangyin Hospital, College of Medicine, Southeast University, Jiangyin, Jiangsu 214400;; 2 Department of Cardiovascular Surgery, Heart Center, General Hospital of Ningxia Medical University, Yinchuan, Ningxia 750004;; 3 Department of Cardiology, The Second People’s Hospital of Panyu, Panyu, Guangdong 511470;; 4 Department of Cardiology, Wuhan Central Hospital, Wuhan, Hubei 430014, P.R. China

**Keywords:** paroxysmal atrial fibrillation, aspirin, warfarin, middle risk, high risk

## Abstract

The aim of the present study was to determine the optimal intensity of anticoagulation therapy in elderly patients with paroxysmal atrial fibrillation (PAF), using aspirin and varied concentrations of warfarin. Elderly patients with PAF (n=1,162) who met the inclusion criteria of the study and were at middle or high-risk of a stroke were investigated. Patients were divided into six groups (four high-risk groups and two middle-risk groups). Patients were treated with aspirin or varied concentrations of warfarin. The primary endpoint events, secondary endpoint events, major bleeding events and minor bleeding events were observed and compared. In high-risk elderly patients, warfarin significantly reduced primary and secondary endpoint events, total primary events and total events compared with aspirin. In middle-risk elderly patients, for all the events warfarin demonstrated no significant difference compared with aspirin. In high-risk patients with PAF, when the concentration of warfarin was adjusted to target international normalized ratio (INR) range 1.7–2.5, the primary and secondary endpoint events, total primary events and total events were significantly lower (P<0.05), compared with aspirin and warfarin at INR 1.2–1.6. When the intensity of warfarin was adjusted to the target INR 2.6–3.0, the primary and secondary endpoint events were significantly lower (P<0.05) compared with aspirin and warfarin INR at 1.2–1.6. This study determined that in high-risk elderly patients with PAF, warfarin is recommended for anticoagulation with an optimal INR range of 1.7–2.5. In patients at a middle-risk of a stroke, aspirin is the recommended treatment as an antithrombotic as results have indicated that there is limited benefit in the use of warfarin.

## Introduction

The famous cardiologist, Eugene Braunwald indicated that atrial fibrillation (AF) has become an epidemic in the 21st century. Therefore, to prevent thromboembolic events in patients with AF antithrombotic therapy has been studied. A study determined that the average rate of ischemic stroke among patients with AF is ∼5% per year (1). However, it has been indicated that warfarin may significantly reduce the incidence of a stroke in patients with AF (2). Clinical trials utilizing warfarin for the prevention of cerebral embolism in patients with AF, have demonstrated that an appropriate anticoagulation intensity directed by international normalized ratio (INR), may be an effective and safe method for the use of warfarin. In addition, the Boston area anticoagulation trial (BATT) determined that low-dose warfarin therapy (INR 2.0–3.0) is highly effective in preventing a stroke in patients with non-rheumatic AF, without an increased risk of major bleeding, and may be a safe treatment with careful monitoring (3). Furthermore, a meta-analysis of five randomized trials of oral anticoagulants (OACs) compared with the control, for the primary prevention of a stroke in patients with non-valvular AF [aspirin versus warfarin standard dose (AFASAK I); aspirin versus warfarin standard dose, age >75 (SPAF II); warfarin versus no treatment (BAATAF); warfarin versus placebo (SPINAF); and Canadian Atrial Fibrillation Anticoagulation (CAFA)], identified that INR 2.0–2.6 provided the lowest risk of bleeding and a stroke. However, if INR>3, the risk of bleeding increased (4). The increase in risk of a stroke was similar between chronic AF and paroxysmal AF (PAF) (5). Studies that have investigated AF anticoagulation strategies in China have been established according to the European or American guidelines. However, a large-scale clinical study has yet to be performed and may confirm an appropriate anticoagulation concentration (INR range) for Chinese elderly patients with AF. Moreover, it is a controversial issue as to whether elderly patients with PAF who have a middle- to high-risk of a stroke, should receive antithrombotic therapy. Additionally, the optimal concentration of warfarin is still unknown. A study has demonstrated that blood coagulation in Asian patients is lower than that of European and American patients; therefore, lower intensity anticoagulation therapy is recommended (6). Thus, large-scale clinical studies on anticoagulation of elderly Chinese patients with PAF, is required to ensure effective and safe treatment with warfarin.

## Patients and methods

### Patients

The present investigation was a prospective, randomized, controlled, parallel and multicentered study. The definition and classification in the American College of Cardiology/American Heart Association Task Force on Practice Guidelines/European Society of Cardiology Committee (ACC/AHA/ESC; 2006) guidelines for the management of patients with AF was followed (7). Patients with PAF were divided into three groups on the basis of stroke risk: low-, middle- and high-risk. Middle-risk patients had one of the following risk factors: age ≥75 years, heart failure, hypertension, diabetes, and left ventricular ejection fraction (LVEF) ≤0.35. High-risk patients had two of the risk factors listed, or one of the following risk factors: previous history of a stroke, transient ischemic attack or thromboembolism.

### Population

Elderly patients aged ≥65 years with PAF were randomly assigned to aspirin or varied concentrations of warfarin for antithrombotic therapy. Patients who met all the inclusion criteria and none of the exclusion criteria were recruited at each center. This included patients aged ≥65 years; had indicated at least two documented AF episodes in the previous six months with a duration of <3 days [confirmed by electrocardiography (ECG) or Holter]; patients that demonstrated palpitations, chest tightness, dizziness and sweating; and patients that were either at a middle or high-risk of a stroke. The exclusion criteria were recorded by the clinicians and determined the eligibility of a patient. The criteria included patients with non-artherosclerosis AF (rheumatic heart disease, cardiomyopathy, hyperthyroidism and electrolyte disturbances); AF due to reversible underlying disease (acute myocardial infarction, acute myocarditis and untreated hyperthyroidism); AF induced by electrophysiological examination, coronary angiography or pacemaker implantation; patients with a recent history of cardiothoracic surgery, gastrointestinal and intracranial bleeding or other bleeding; severe liver or renal dysfunction; cancer or blood disease; and acute inflammation of the respiratory tract or urinary tract. This study had a total of 1,472 patients. The local ethic committee approved the study protocol and written informed consent was obtained from all patients.

### Baseline evaluation and grouping

**Figure 1.**
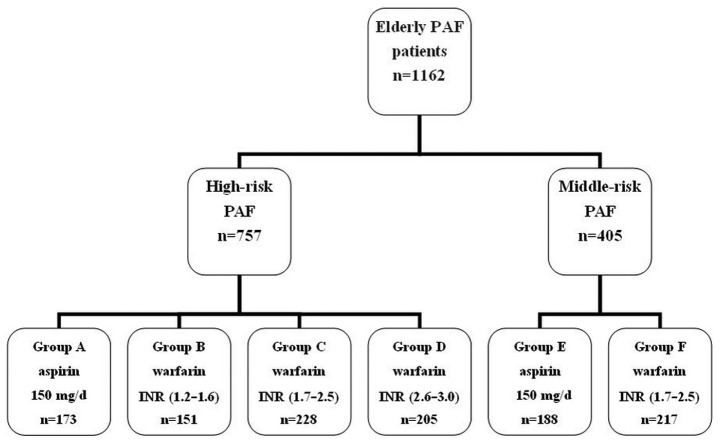
Patient grouping. PAF, paroxysmal arterial fibrillation; INR, international normalized ratio.

Prior to the randomization, baseline characters of the patients were evaluated. This included their medical history, a physical examination, 12-lead ECG, 24-h Holter, assay of D-dimer and prothrombin times, vascular ultrasound of the carotid and leg arteries and an echocardiogram. In addition, all patients were computer randomized using SPSS software, version 13.0 (SPSS, Inc. Chicago, Il, USA) into four high-risk groups and two middle-risk groups; Group A: high-risk, administered 150 mg/day aspirin; Group B: high-risk, administered 1.875 mg/day warfarin, then the dose was adjusted to maintain a target INR range of 1.2–1.6; Group C: high-risk, administered 2.5 mg/day warfarin, then the dose was adjusted to a target INR range of 1.7–2.5; Group D: high-risk, administered 2.5 mg/day warfarin, then dose was adjusted to a target INR range of 2.6–3.0. The two middle-risk groups were Group E: administered 150 mg/day aspirin; and Group F: administered 2.5 mg/day warfarin, then dose was adjusted to a target INR range of 1.7–2.5 (Fig. 1). Prior to enrollment in this study, the target INR range was achieved in the groups administered with warfarin (Groups B, C, D and F).

### Patient follow-up

Patients were seen in the institutional outpatient clinics one, two, three and six months following treatment, then follow-up every 3–6 months and then as required. Routine tests including prothrombin time and INR were performed. In addition, various events were recorded including; primary endpoint events (death, stroke, pulmonary embolism) and secondary endpoint events [acute myocardial infarction, lacunar infarction, transient ischemic attack (TIA), asymptomatic stroke and peripheral arterial embolism], major bleeding events (cerebral hemorrhage, gastrointestinal bleeding) and minor bleeding events (skin, mucous membrane and gums bleeding, hematuria). In addition, the total primary events (primary endpoint events and major bleeding events), total secondary events (secondary endpoint events and minor bleeding events) and total events (total primary events and total secondary events) were calculated. All the events were evaluated synergistically by a cardiologist and neurologist.

### Statistical analysis

Statistical analysis of the data was performed on SPSS software, version 13.0 (SPSS Inc, Chicago, Il, USA). All means were expressed with SD for continuous variables when normally distributed and comparisons were evalutated using the Student’s t-test or Mann-Whitney U-test, as appropriate. Categorical variables were expressed as numbers (with %), differences between groups were analysed using Pearson’s χ^2^ test or Fisher’s exact test. P<0.05 is considered to indicate a statistically significant difference.

## Results

### Demographic and clinical characteristics

The demographic and clinical characteristics of the patients in this study were identical in the high- or middle-risk groups (Table I). The total patients at the end of the study was 1,472. There were 310 invalid cases as they had met one of the exclusion criteria including; <6 months treatment duration; patients were unable to follow the instructions, refused to continue the drugs, or did not monitor the INR regularly; a new onset of other diseases was indicated during the follow-up period, including rheumatic heart disease, idiopathic cardiomyopathy, encephalitis, hyperthyroidism, cancer, blood diseases or the patient needed surgery; and complication of severe liver or renal dysfunction during the follow-up period. The total 1,162 cases (females; 458) were regarded as valid cases, the mean (SD) age of these patients was 72.5±4.4 years (range; 65–78 years). Between the four high-risk groups (A, B, C and D), or between the two middle-risk groups (E and F), age, sex distribution and smoking status were approximately identical. There were no significant differences in the history of hypertension, diabetes, hyperlipidemia, stroke, TIA, myocardial infarction, peripheral vascular thrombosis, pulmonary embolism and other diseases. The use of ACEI/ARB and β-blockers were similar between Groups A, B, C and D, or Groups E and F. The incidence of LVEF<35%, follow-up time and INR value prior to treatment also indicated no significant differences (P>0.05).

### Comparison of events in high-risk patients

In high-risk patients, the primary and secondary endpoint events in Groups C and D were significantly lower (P<0.05) compared with those in Groups A and B (Table II). However, there were no significant differences between Groups A and B or between Groups C and D (P>0.05). The major and minor bleeding events among the four groups (A, B, C and D) indicated no significant differences (P>0.05). However, Groups C and D indicated a higher number of major bleeding events compared with those in Groups A and B. In addition, the total primary events in Group C were significantly lower (P<0.05) compared with those in Groups A and B (P<0.05). However, there were no significant differences between Groups A, B, D or between Groups C and D (P>0.05). Total secondary events in Group C were significantly lower than those in Group B (P<0.05); however, there were no significant differences between Groups A, B and D or between Groups C, A and D (P>0.05). The total events in Group C were significantly lower than those in Groups A and B (P<0.05); however, there were no significant differences between Groups A, B and D or between Groups C and D (P>0.05).

### Comparison of aspirin with warfarin in high-risk or middle-risk patients

In high-risk patients with PAF, warfarin significantly reduced the primary and secondary endpoint events, total primary events and total secondary events (P<0.05) compared with aspirin, whereas major and minor bleeding events were not significantly different (P>0.05; Table III). In middle-risk patients with PAF, there were no significant differences in any events between the aspirin or warfarin groups (Table IV).

## Discussion

AF is frequently divided into three categories: PAF, persistent AF and chronic AF (8). Chronic AF indicates the long-term presence of symptoms (seven days-years), and is also known as permanent AF. PAF is a recurrent AF and commonly lasts <7 days and may be inhibited without treatment for the majority of cases. If AF continues >48 h (∼7 days), known as persistent AF, the patient is unlikely to revert back to normal without treatment, and may regain a normal rhythm with cardioversion (9).

At present, it has not been concluded whether patients with PAF have a similar risk factor for developing ischemic stroke as patients with chronic AF. The Framingham Heart Study (10) demonstrated a 5.6-fold increased risk for the development of embolism in patients with non-rheumatic AF when compared with controls. Non-rheumatic AF is thought to be responsible for a large percentage of strokes, as it is present in ∼15–20% of cerebrovascular accidents of ischemic origin. In addition, the risk of thromboembolic events in patients with non-rheumatic AF was ∼5% per year. Previously, it has been determined that PAF and persistent AF may have a similar risk for developing ischemic stroke. A study has indicated that chronic AF carries a risk of 6% per year for the development of thromboembolic events, this is higher than the 2–3% risk per year in paroxysmal AF (11). A meta-analysis of five randomized controlled trials demonstrated that type (paroxysmal or chronic) and duration of AF has no significant effect on the incidence of a stroke. The risk of embolism is higher immediately following the onset of AF, during the first year of chronic AF or following the early conversion to a sinus rhythm (1). Elderly patients with AF that were more likely to suffer cerebrovascular accidents, represented 6.7% of the total number in the 50-to-59-year-old group and 36.2% in the 80-to-89-year-old group (12).

It is important to study antithrombotic therapy in elderly patients due to the diversity of individuals. According to ACC/AHA/ESC 2006 Guidelines for the Management of Patients with AF (7), the dose of oral warfarin may be adjusted to maintain the target INR 2.0–3.0; however, the guidelines are based on the American and European population. In comparison, the Asian populations tend to have lower coagulation activation, and it has been suggested they require a lower intensity of anticoagulation therapy (6).

The present study lasted for >5 years and it was a single-blind, randomized and multicentered study. Elderly patients (n=1,472) with PAF who demonstrated a middle-high risk of having a stroke, were used in this study. From the 1,472 cases, 310 patients were invalid due to various reasons, leaving 1,162 cases which were analyzed. In this study, the total primary events (primary endpoint event and major bleeding events) was the major indicator for optimal antithrombotic therapy (aspirin or warfarin). In Group C (high-risk patients, 2.5 mg/day warfarin then the dosage was adjusted to a target INR 1.7–2.5) the primary and secondary endpoint events, total primary events and total events were all significantly lower compared with Group A (high-risk, administered 150 mg/day aspirin) and Group B (high-risk, administered 1.875 mg/day warfarin then the dose was adjusted to a target INR 1.2–1.6; P<0.05). In addition, the primary and secondary endpoint events were significantly lower in Group D (high-risk, administered 2.5 mg/day warfarin then the dose was adjusted to a target INR 2.6–3.0) compared with Groups A and B (P<0.05). In Group C, the total primary events (the major indicator for the optimal antithrombotic therapy) was significantly less than those in Groups A and B. However, no significant difference was identified in Group D, possibly due to a high number of major bleeding events (13 cases). Therefore, the increased anticoagulation (INR, 2.6–3.0) in Group D did not benefit the patients. Furthermore, there were no significant differences between Groups A and B in any event, which indicated that low intensity anticoagulation (warfarin, INR 1.2–1.6) had no increased benefit compared with aspirin. This study concluded that the optimal INR range is 1.7–2.5 for Chinese elderly patients with PAF.

Hylek *et al* confirmed that anticoagulant prophylaxis of ischemic stroke is effective at INRs of ≥2.0, in patients with AF (13). The risk of a stroke increased steeply at INRs of <2.0. At a decreasing INR, the adjusted odds ratio determining the risk factor of a stroke increased; INR of 1.7, ratio of 2.0; INR of 1.5, ratio of 3.3 and INR of 1.3, ratio of 6.0. This is in accordance with the results of the present study. A prospective multicentered, randomized, Japanese non-valvular AF study (6) showed that a high concentration of warfarin anticoagulation treatment (INR 2.2–3.5) significantly increased bleeding events compared with a lower intensity of warfarin (INR 1.5–2.1). This indicated that blood coagulation in Asian individuals is lower compared with European and American individuals. A further study (14) in China also demonstrated that in elderly patients with PAF, the anticoagulation intensity of INR 1.7–2.5 is safe and effective, a finding that is supported by the results from the present study.

The present study demonstrated that in high-risk patients with PAF, warfarin reduced a larger number of primary and secondary endpoint events, total primary events and total events when compared with aspirin. In middle-risk patients with PAF, Group F (warfarin) demonstrated a higher number of secondary endpoint events when compared with Group E (aspirin); however, these were not the major indicators. Therefore, in clinical practice, it is necessary to analyze elderly patients with PAF based on their risk of a stroke. This study concluded that warfarin may be used in high-risk elderly patients with a target INR of 1.7–2.5. However, in patients at middle-risk of a stroke, aspirin is the preferred treatment of PAF and the use of warfarin is limited.

## Figures and Tables

**Table I.** Characteristics of the patients.

Variable (n)	Group A (n=173)	Group B (n=151)	Group C (n=228)	Group D (n=205)	Group E (n=188)	Group F (n=217)
Age (years)	72.4±4.9	73.1±4.7	72.8±4.5	72.2±4.9	72.8±4.5	71.9±4.3
Male (%)	108 (62.4)	92 (60.9)	141 (61.8)	123 (60.0)	114 (60. 6)	126 (58.1)
Smoking (%)	65 (37.6)	53 (35.1)	85 (37.3)	73 (35.6)	67 (35.6)	80 (36.8)
Hypertension (%)	72 (41.6)	61 (40.4)	91 (39)	82 (40.0)	75 (39.9)	86 (39.6)
Diabetes (%)	65 (37.6)	57 (37.7)	83 (36.4)	75 (36.6)	69 (36.7)	83 (38.2)
Hyperlipidemia (%)	51 (29.5)	45 (29.8)	66 (28.9)	60 (29.3)	54 (28.7)	63 (29.0)
Prior stroke (%)	38 (21.9)	34 (22.5)	49 (21.5)	44 (21.5)	0	0
Prior TIA (%)	25 (14.5)	21 (13.9)	32 (14.0)	29 (14.1)	0	0
Prior AMI (%)	16 (9.2)	12 (8.0)	20 (8.8)	18 (8.8)	10 (5.3)	12 (5.5)
LVEF<35% (%)	18 (10.4)	14 (9.3)	23 (10.1)	20 (9.8)	0	0
Prior peripheral vascular thrombosis	6 (3.5)	5 (3.3)	8 (3.5)	7 (3.4)	0	0
Prior pulmonary embolism (%)	10 (5.8)	9 (5.9)	13 (5.7)	11 (5.4)	0	0
ACEI/ARB (%)	102 (59.0)	90 (59.6)	134 (58.8)	124 (60.5)	110 (58.5)	126 (56.7)
β-blockers (%)	68 (39.3)	60 (39.7)	88 (38.6)	80 (39.0)	74 (39.4)	85 (39.2)
Follow-up (months)	50.7±13.8	51.3±12.8	51.6±13.8	51.3±12.9	50.7±11.6	51.4±12.2
INR prior to treatment	0.86±0.12	0.88±0.15	0.84±0.17	0.87±0.12	0.81±0.11	0.79±0.10

TIA, transient ischemic attack; AMI, acute myocardial infarction; LVEF, left ventricular ejection fraction; ACEI/ARB, angiotensin-converting enzyme inhibitors/angiotensin II receptor antagonists; INR, international normalized ratio.

**Table II.** Comparison of events in high-risk patients.

Variable (n)	Group A (n=173)	Group B (n=151)	Group C (n=228)	Group D (n=205)
Primary endpoint events	19	14	9^a,b^	8^a,b^
Death	6	5	3	4
Ischemic stroke	10	8	4	2
Pulmonary embolism	3	3	2	1
Secondary endpoint events	30	25	19^a,b^	16^a,b^
Acute myocardial infarction	3	4	4	4
Lacunar infarction	8	6	5	3
TIA	7	6	4	3
Peripheral arterial embolism	6	4	2	3
Asymptomatic stroke	6	5	4	3
Major bleeding events	5	5	7	13
Cerebral hemorrhage	2	3	4	9
Gastrointestinal bleeding	3	3	3	4
Minor bleeding events	11	14	20	24
Hematuria	5	6	9	9
Skin, mucous membrane and gums bleeding	6	8	11	15
Total primary events	24	20	16^a,b^	21
Total secondary events	41	39	39^b^	40
Total events	65	56	55^a,b^	61

a P<0.05 compared with Group A;

b P<0.05 compared with Group B. TIA, transient ischemic attack.

**Table III.** Comparison of aspirin (Group A) with warfarin (Group C) in high-risk patients.

Variable (n)	Group A (n=173)	Group C (n=228)	χ^2^	P-value
Primary endpoint event	19	9	7.496	0.006^a^
Death	6	3		
Ischemic stroke	10	4		
Pulmonary embolism	3	2		
Secondary end points	30	19	7.441	0.006^a^
Acute myocardial infarction	3	4		
Lacunar infarction	8	5		
TIA	7	4		
Peripheral arterial embolism	6	2		
Asymptomatic stroke	6	4		
Major bleeding event	5	7	0.011	0.917
Cerebral hemorrhage	2	4		
Gastrointestinal tract bleeding	3	3		
Minor bleeding	11	20	0.803	0.370
Hematuria	5	9		
Skin and mucous membrane bleeding gums	6	11		
Total primary events	24	16	5.148	0.023^a^
Total secondary events	41	39	2.678	0.102
Total events	65	55	8.485	0.004^a^

a P<0.05 indicated a statistically significant difference between Groups A and C. TIA, transient ischemic attack.

**Table IV.** Comparison of aspirin (Group E) with warfarin (Group F) in middle-risk patients.

Variable (n)	Group E (188)	Group F (217)	χ^2^	P-value
Primary endpoint event	14	7	3.651	0.056
Death	4	3		
Ischemic stroke	7	3		
Pulmonary embolism	3	1		
Secondary end points	19	11	3.727	0.054
Acute myocardial infarction	3	3		
Lacunar infarction	7	3		
TIA	4	2		
Peripheral arterial embolism	1	1		
Asymptomatic stroke	4	2		
Major bleeding event	3	5	0.261	0.069
Cerebral hemorrhage	1	3		
Gastrointestinal tract bleeding	2	2		
Minor bleeding	8	12	0.349	0.555
Hematuria	4	6		
Skin and mucous membrane bleeding gums	4	6		
Total primary events	17	12	1.87	0.172
Total secondary events	27	23	1.318	0.251
Total events	44	35	3.396	0.065
